# Rockwool-Based Fertigation Enhances Tea Plant Growth While Mitigating Soil N_2_O Emissions

**DOI:** 10.3390/plants15121862

**Published:** 2026-06-16

**Authors:** Zhongqian Wang, Bo Fan, Qiufang Xu, Shuai Shao

**Affiliations:** College of Environmental and Resource Sciences, Zhejiang A&F University, Hangzhou 311300, China; wangzq@stu.edu.cn (Z.W.); bfan0928@zafu.edu.cn (B.F.)

**Keywords:** rockwool, fertigation, N_2_O emissions, tea plantations

## Abstract

Mitigating nitrous oxide (N_2_O) emissions from cropland soils is a pressing challenge for climate change mitigation. This study evaluated rockwool-based fertigation (RF) in reducing N_2_O emissions from tea plantations. A 17-month field experiment was conducted comparing RF with conventional surface fertilization (CK), measuring tea plant biomass, new tea shoots yield, new tea shoots quality indices, soil N_2_O fluxes, physicochemical properties, and nitrogen (N)-cycling functional genes across different soil layers. Results showed that RF treatment significantly increased the aboveground pruning biomass of tea plants, suggesting that RF promotes tea plant growth. The RF treatment showed lower N_2_O fluxes and cumulative N_2_O emissions within 90 days post-fertilization across the tea-growing season compared with CK, demonstrating that RF effectively mitigates N_2_O emissions from tea plantation soils. Random forest analysis further revealed that the RF-induced vertical redistribution of nutrients and N-cycling functional genes was the primary driver of N_2_O mitigation. Our findings demonstrate that RF is an effective dual-benefit strategy that simultaneously enhances tea plant productivity and mitigates N_2_O emissions by reshaping soil biogeochemical processes and their spatial distribution.

## 1. Introduction

Nitrous oxide (N_2_O) is the third most significant anthropogenic greenhouse gas. It plays a pivotal role in both the global nitrogen (N) cycle and climate change [1]. Cropland soils constitute a major source, contributing over 3 Tg of N_2_O-N annually and accounting for more than 60% of total anthropogenic emissions [2]. Notably, approximately half of these emissions are directly linked to synthetic N fertilizer application [3]. Therefore, N management strategies can simultaneously influence multiple environmental outcomes, including greenhouse gas emissions and trace element mobility [4]. Developing and implementing effective mitigation strategies to curb fertilizer-derived N_2_O emissions is imperative for reducing the agricultural greenhouse gas footprint. Previous studies have demonstrated that deep placement of fertilizers can significantly reduce N_2_O and NH_3_ emissions [5], as well as overall N losses by restricting substrate availability for nitrification and denitrification in surface soils [6,7]. Consequently, advancing deep fertilization techniques represents a promising pathway toward sustainable N_2_O mitigation in cropping ecosystems.

Fertigation, which integrates water and fertilizer application into a unified delivery system, enhances water use efficiency and crop yields by synchronizing resource supply with crop demand [8,9]. Optimizing the synchrony between water and nutrient supply is critical for reducing environmental losses, as demonstrated in studies of irrigation scheduling and soil water balance in other cropping systems [9]; thus, it is also substantially mitigating soil N_2_O emissions [10]. For instance, subsurface drip irrigation at a depth of 30 cm reduced N_2_O emissions by nearly 29% compared with surface fertilization [11], and other studies have identified 25 cm as the optimal depth for emission mitigation [12,13]. This phenomenon is attributed to depth-dependent biogeochemical processes. N_2_O production predominantly occurs in surface soils and declines sharply with depth, often becoming negligible below 30 cm [14]. This reduction correlates with a higher relative abundance of N_2_O-reducing bacteria in deeper soil layers [15]. Furthermore, N_2_O generated at depth must traverse a longer diffusion path to reach the atmosphere [16]; this prolonged transit increases its residence time within the soil matrix, thereby enhancing the probability of complete reduction to N_2_ by denitrifying microorganisms [17,18].

However, a critical limitation of conventional deep-fertigation systems is their inability to retain water and nutrients at the target zone. Fertilizer solutions delivered via emitters tend to disperse rapidly, resulting in inefficient crop uptake and potential environmental losses [19]. To address this, we employed rockwool—a fibrous inorganic material derived from natural basalt—to develop a novel rockwool-based fertigation (RF) system. This technology integrates rockwool with deep placement, serving as a long-term carrier for water and nutrients [7]. This system delivers fertilizer solutions directly to a buried rockwool module, which leverages its high water-holding and cation-exchange capacities to regulate the storage and controlled release of nutrients, thereby creating an optimal root zone microenvironment [20]. Nevertheless, the effects of RF on soil N_2_O emissions and their underlying relationships with soil physicochemical parameters and functional microbes remain poorly understood.

Tea (*Camellia sinensis* L.) is one of the most widely consumed beverages worldwide, and plantation areas have been expanding rapidly [21]. To sustain high yields and premium leaf quality, these plantations are routinely supplied with excessive N fertilizer, predominantly via surface broadcast application [22]. This intensive management practice promoted N_2_O formation in soils via nitrification and denitrification [23]. Consequently, tea plantations have emerged as globally significant anthropogenic N_2_O sources. While deep fertigation has shown promise in reducing N_2_O emissions in other cropping systems [24,25], its efficacy in tea plantations remains unexplored. In particular, the interplay between RF-induced shifts in soil physicochemical properties and the responses of N-cycling functional microbial communities—and how these interactions collectively govern N_2_O dynamics in tea plantation soils—has yet to be elucidated.

To address this knowledge gap, we conducted a 17-month field experiment (from October 2021 to February 2023) in a tea plantation to compare the RF system with conventional surface fertilization. Soil N_2_O fluxes were systematically monitored using the static closed-chamber method [25,26], and the abundances of key N-cycling functional genes (AOA, AOB, *nirK*, *nirS*, and *nosZ*) were quantified via quantitative PCR [27,28]. The objectives of this study were to (i) quantify the response of soil N_2_O emissions to the RF treatment and (ii) elucidate the underlying mechanisms, with particular emphasis on the interplay between RF-induced shifts in soil physicochemical properties and the dynamics of N-cycling functional genes abundance. We hypothesized that (1) RF would substantially mitigate cumulative N_2_O emissions, driven by alterations in soil physicochemical properties and the restructuring of N_2_O-related functional microbial communities; and (2) the N_2_O mitigation potential of RF would become increasingly pronounced with extended deployment, as the rockwool module progressively establishes a stable and favorable root zone microenvironment.

## 2. Results

### 2.1. Environmental Conditions and Soil Properties

**Figure 1:**
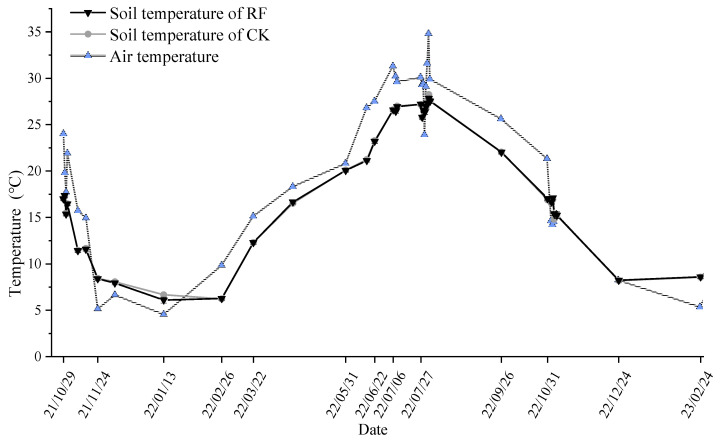
Seasonal variations in air and soil temperature under different treatments from October 2021 to February 2023. CK, conventional surface fertilization; RF, rockwool-based fertigation.

**Figure 2:**
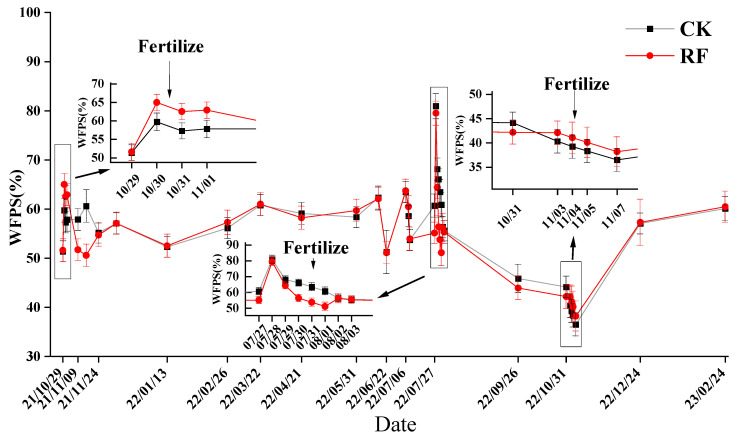
Seasonal variations in WFPS under different treatments from October 2021 to February 2023. CK, conventional surface fertilization; RF, rockwool-based fertigation.

Throughout the experimental period, soil temperature demonstrated distinct seasonal variations, peaking at an average of 28 °C in August and dipping to a low of 6.3 °C in February (Figure 1). No significant differences in soil temperature were observed between the two treatments. Soil WFPS also exhibited seasonal fluctuations, reaching a maximum of over 80% in August and a minimum of approximately 40% in November 2022 (Figure 2). Notably, WFPS decreased to around 50% in September 2022, coinciding with an extreme heat and drought event in the summer of 2022. For the remainder of the study, WFPS remained relatively stable, and no significant treatment effects were detected at any time point.

The RF treatment significantly enhanced tea plant growth, as evidenced by 6.45% (*p* < 0.05) increase in aboveground pruning biomass compared to the CK (10.59 ± 0.34 vs. 9.87 ± 0.20 t ha^−1^, Table 1). Additionally, the RF treatment significantly altered the vertical distribution of soil properties (Table 2). In the 0–10 cm soil layer, concentrations of SOC, TN, MBC, NH_4_^+^-N, and NO_3_^−^-N were significantly lower under the RF treatment. Conversely, in the deeper soil layers (10–20 cm and 20–40 cm), the RF treatment resulted in significant increases in SOC, TN, MBC, MBN, and NO_3_^−^-N concentrations.

### 2.2. Tea Plant Biomass, Bud Yield and Bud Quality Indices

Tea yield and quality indices (amino acids, tea polyphenols, total nitrogen, total phosphorus, and total potassium) of tender leaves showed no significant differences between the RF and CK treatments. However, the aboveground pruning biomass of tea plants under the RF treatment was significantly increased by 6.45% compared to the CK (Table 3).

### 2.3. Soil N_2_O Fluxes and Cumulative Emissions

**Figure 3:**
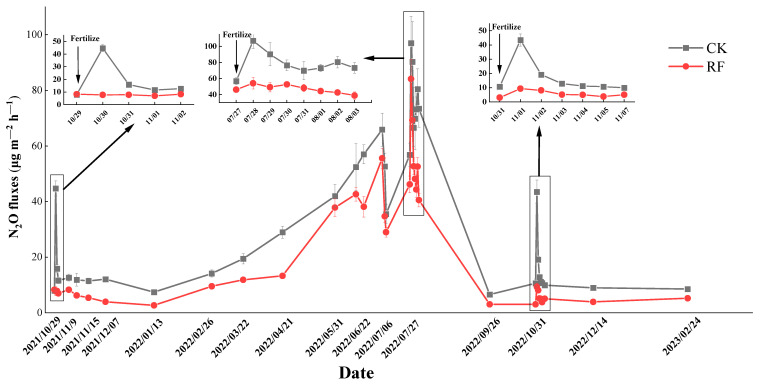
Effect of rockwool-based fertigation on soil N_2_O flux. CK, conventional surface fertilization; RF, rockwool-based fertigation.

The dynamics of N_2_O fluxes were similar for both the RF and CK treatments (Figure 3). Higher N_2_O fluxes were observed from July to August after fertilization than from October to November, coinciding with higher soil temperature and WFPS (Supplemental Figures S1 and S2). The RF treatment exhibited lower N_2_O fluxes than the CK treatment throughout the tea-growing season, particularly in the first week after fertilization, while the CK treatment showed a transient N_2_O emission peak after fertilization.

**Figure 4:**
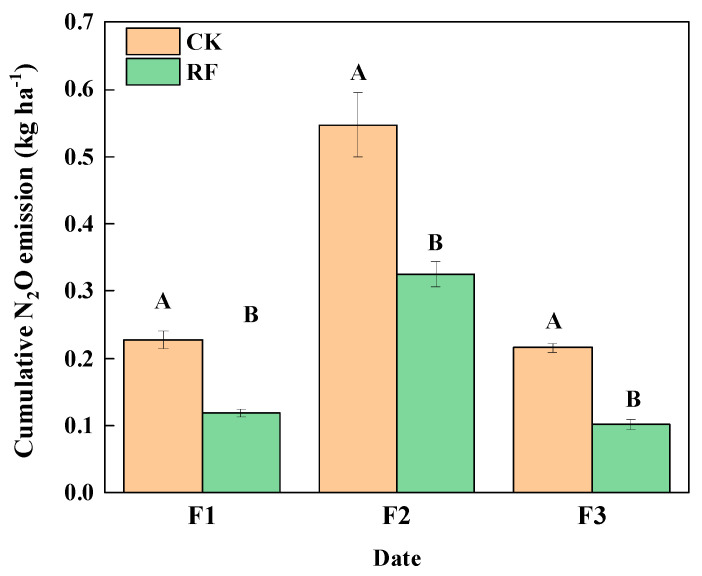
Effect of rockwool-based fertigation on soil cumulative N_2_O emission. CK: conventional surface fertilization; RF: rockwool-based fertigation. F1: The first 90 days after fertilization from 31 October 2021 to 26 January 2022. F2: The second 90 days after fertilization from 27 July 2022 to 24 October 2022. F3: The second 90 days after fertilization from 31 October 2022 to 28 January 2023. Different capital letters indicate significant differences among the 90 days after fertilization. Error bars represent standard errors of the mean (SE, *n*  =  6).

**Figure 5:**
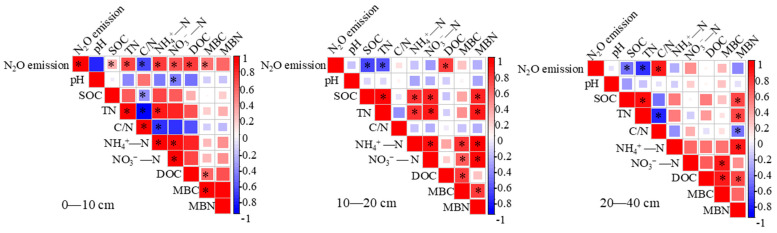
Spearman correlation analysis between soil N_2_O emission and physicochemical properties. SOC, soil organic carbon; TN, total nitrogen; C/N, ratio of soil organic carbon to total nitrogen; MBC, microbial biomass carbon; MBN, microbial biomass nitrogen; and DOC, dissolved organic C. * represent the significant difference(*p* < 0.05).

Cumulative N_2_O emissions within 90 days following each of the three fertilization events were consistently and significantly lower in the RF treatment compared to the CK treatment (*p* < 0.05, Figure 4). Additionally, the difference in cumulative N_2_O emissions between the RF and CK treatments from October 2022 to January 2023 was significantly higher than that from October 2021 to January 2022. The percentage reduction in cumulative N_2_O emissions within 90 days following the third fertilization was significantly higher in the RF treatment compared to the first two fertilization events (*p* < 0.05, Figure 5).

### 2.4. Abundances of N-Cycling Functional Genes

The RF treatment significantly altered the vertical distribution of N-cycling functional genes (Supplemental Figures S3 and S4). In the 0–10 cm soil layer, the RF treatment led to substantial decreases in the abundances of both nitrification and denitrification genes. Compared to CK, the gene abundances of ammonia-oxidizing archaea (AOA) and ammonia-oxidizing bacteria (AOB) were reduced by 58.3% and 70.1%, respectively. Similarly, the abundances of the denitrification genes *nirS*, *nirK*, and *nosZ* were significantly lower under RF, with reductions of 38.9%, 60.8%, and 50.3%, respectively.

Conversely, in the 10–20 cm layer, the RF treatment markedly increased the abundance of these genes. The AOA and AOB gene abundances increased by 48.2% and 477.3%, respectively, while *nirS*, *nirK*, and *nosZ* abundances increased by 34.1%, 60.6%, and 72.4%, respectively. In the deepest layer (20–40 cm), no significant differences were observed for most genes, with the exception of a 61.5% increase in *nirK* abundance under the RF treatment.

### 2.5. Dependence of N_2_O Emissions on Biochemical Properties

**Figure 6:**
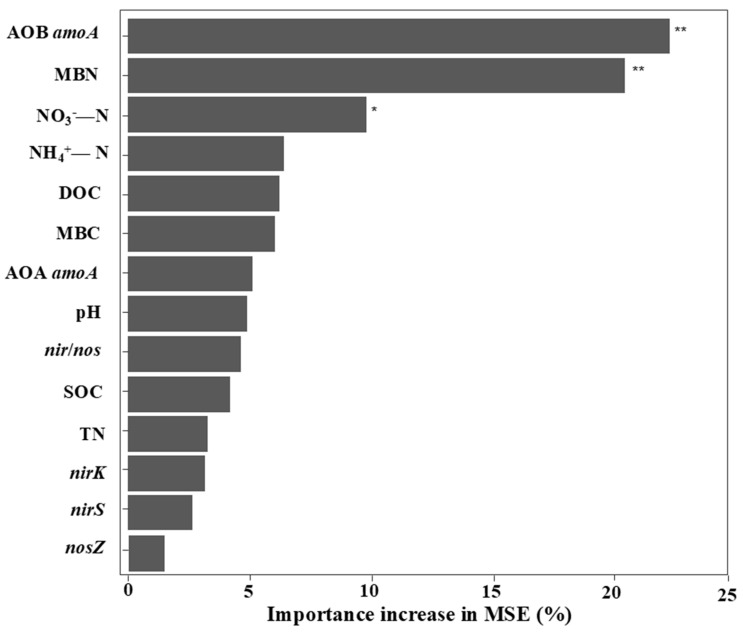
Random forest regression analysis between N_2_O emissions and soil factors in RF treatment. SOC, soil organic carbon; TN, total nitrogen; C/N, ratio of soil organic carbon to total nitrogen; MBC, microbial biomass carbon; MBN, microbial biomass nitrogen; and DOC, dissolved organic C. %IncMSE represents the percentage increase in mean squared error, indicating variable importance. * and ** represent the significant difference (*p* < 0.05) and (*p* < 0.01) respectively.

Correlation analysis between N_2_O emissions and soil physicochemical properties is shown in Figure 5. In the 0–10 cm layer, N_2_O emissions were positively correlated with TN, NH_4_^+^-N, NO_3_^−^-N, MBC, MBN, and DOC concentrations, but negatively correlated with soil pH and the C/N ratio (*p* < 0.05). However, N_2_O emissions showed negative correlations with SOC, TN concentrations in the 10–20 cm and 20–40 cm soil layers. And it also exhibited a significant positive correlation with DOC concentration in the 10–20 cm soil layers, and significant positive correlation with C/N ratio in the 20–40 cm soil layers. Random forest analysis further identified that the key influential variables for N_2_O emissions in the RF treatment were MBN, AOB, and NO_3_^−^-N concentration (Figure 6). 

## 3. Discussion

### 3.1. Evidence and Key Mechanisms of N_2_O Emission Reduction

Although the soil N_2_O fluxes of both RF and CK treatments showed a similar pattern during the entire monitoring period (Figure 3), the RF treatment consistently led to significantly lower cumulative N_2_O emissions compared to the CK treatment. This result validates our first hypothesis, demonstrating the effectiveness of RF in mitigating N_2_O emissions from tea plantation soils. Some previous studies have recommended that drip irrigation be used to reduce N_2_O emissions [24]. The significant reduction in N_2_O emissions in the RF treatment can be mainly attributed to its strategic alteration of the vertical distribution of soil nutrients, which directly impacts N_2_O production and consumption dynamics within the soil profile.

Our results clearly showed that the RF treatment significantly decreased the concentrations of available nutrients in the surface soil (0–10 cm) while increasing them in the subsoil (10–40 cm). This improved subsoil nutrient availability likely contributed to the significant increase in above-ground tea plant biomass (Table 3). The enhanced tea plant growth under RF may create positive biophysical feedback on soil processes, including increased root-derived carbon inputs that further stimulate microbial activity and soil organic matter formation [29]. These additional carbon substrates fuel microbial growth, leading to higher SOC stocks in deeper layers [30,31].

The changes in soil chemical properties also directly led to a vertical restructuring of the N-cycling microbial communities (Supplemental Figure S2). Most notably, the RF treatment shifted the “hotspot” of N-cycling activity from the surface soil to the subsoil. This was evidenced by significantly lower abundances of key nitrification (AOA, AOB) and denitrification (*nirS*, *nirK*, *nosZ*) genes in the 0–10 cm layer, along with a significant increase in these genes within the 10–20 cm layer. This microbial shift is mainly driven by the resource stratification induced by RF. In the subsoil (10–20 cm), the targeted nutrient delivery created an enriched zone with high substrate availability (especially DOC) and a more favorable microenvironment, fostering larger microbial populations. Although nitrifier abundances are typically highest in well-aerated surface soils and decline with depth due to oxygen limitation, the RF treatment overcame this physical advantage by creating a severe substrate limitation in the surface layer.

Crucially, this vertical shift in the microbial “hotspot” directly translates into an effective N_2_O mitigation strategy. The RF system takes advantage of the soil’s natural mitigation pathway by simultaneously cutting off substrate supply to the primary emission zone (0–10 cm). Although the stimulated microbial activity in the 10–20 cm layer may locally increase N_2_O production, the longer diffusion path from this depth ensures that most of the newly produced N_2_O is consumed during its upward migration through the soil matrix. Previous research indicates that the vast majority of N_2_O originates from surface soils (0–15 cm), with emissions decreasing sharply with depth and becoming negligible below 30 cm [32]. This pattern is driven by two main factors: (1) deeper soil layers have a higher relative abundance of N_2_O-reducing bacteria within the denitrifier community [10], and (2) N_2_O produced at depth has to travel a longer diffusion path to the atmosphere.

Based on the random forest analysis, MBN, AOB, and NO_3_^−^-N were identified as the most influential factors. This indicates that the reduction in N_2_O emissions after applying soluble ammonium N fertilizer in the RF treatment is likely due to the transport of nutrients into deeper soil layers. As a result, microbial nitrogen assimilation decreased throughout the entire soil profile. Moreover, nitrification was suppressed, especially the oxidation of ammonia (NH_3_) to nitrite (NO_2_^−^), which subsequently hindered NO_3_^−^-N production. The lower NO_3_^−^-N content in the bulk soil profile may be the key mechanism for the RF treatment to mitigate N_2_O emissions. The net effect of this spatial decoupling of production and emission is a significant decrease in total N_2_O emissions compared to conventional surface fertilization. In addition to microbial pathways, abiotic processes such as iron-catalyzed Fenton-like reactions may contribute to N_2_O consumption in iron-rich acidic tea plantation soils [33,34].

### 3.2. Long-Term Sustainability of the RF Treatment

We also found that the reduction ratio of cumulative N_2_O emissions increased with the RF deployment time, which is in line with our second hypothesis and demonstrates the long-term sustainability of the RF treatment. Upon the commissioning of the RF system, it will have contributed to the sustained mitigation of N_2_O emissions. The high porosity and water-retention capacity of rockwool may also have optimized soil hydrological conditions, potentially reducing localized anaerobic microsites that are favorable for denitrification. Additionally, the stabilized RF system likely improved the spatiotemporal synchrony between soil mineral N supply and crop root uptake, thereby reducing the substrate available for N_2_O-forming microbes during successive fertilization events.

The effectiveness of this approach is further emphasized by the characteristics of the surface soil as an emission hotspot. The surface layer has the highest microbial activity, fueled by greater oxygen availability and labile carbon sources such as DOC [35,36]. Under conventional fertilization (CK), this led to significantly higher abundances of AOA and AOB, driving intense nitrification and contributing to elevated N_2_O fluxes [37]. Our random forest analysis identified SOC and DOC in the 0–10 cm layer as the most powerful predictors of N_2_O emissions, confirming that this layer is the dominant source under standard practices. Moreover, the shorter diffusion path from the surface means that a larger proportion of the produced N_2_O can escape to the atmosphere before being reduced.

## 4. Materials and Methods

### 4.1. Study Site and Management Practices

The field experiment was conducted at a long-term RF experimental site located at the Lingfengsi Forest Farm, Anji County, Zhejiang Province, China (30°28′ N, 119°24′ E). The region experiences a subtropical monsoon climate with a mean annual temperature (MAT) of 16.1 °C and mean annual precipitation (MAP) of 1431 mm. The site is situated on a south-facing slope (~15°) at an elevation of 80 m. The soil at the site is classified as Ferrasol derived from sedimentary parent material [38].

The study involved a 12-year-old tea plantation of *Camellia sinensis* cv. Baiye 1, a representative cultivar of Anji white tea. The tea plants were planted in rows with a spacing of 90 cm. Standard field management practices included annual tea leaf plucking (from February to March), pruning, and fertilization. On 6 July 2022, the tea plants were uniformly pruned to a height of 40 cm, with the pruning residues left on the soil surface. The base fertilizer was applied in late October, followed by the topdressing in July. The water-soluble compound fertilizer (N:P_2_O_5_:K_2_O = 18:4:19) was applied at a rate of 556 kg ha^−1^, consistent with local fertilization practices, which is equivalent to 100 kg N ha^−1^. The initial soil properties are summarized in Table 1.

### 4.2. Experimental Design and System Installation

The RF system was installed in May 2021. Six inter-row spaces (each 90 cm wide and 8 m long) with uniform growth and topography were selected as experimental plots. To prevent cross-interference, adjacent plots were separated by one untreated tea row. A randomized complete block design was employed, with six replicates for both the RF and control treatments.

**Figure 7:**
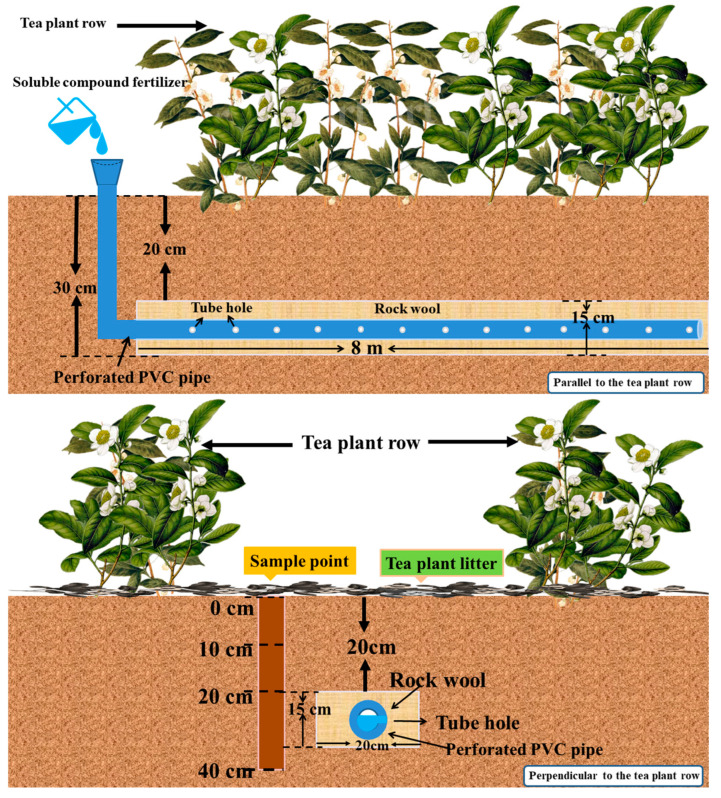
Schematic layout of rockwool-based fertigation device. The water tank is located at the highest point of the field. It is connected to the water pipes of each tea plant row through a main water pipe for water distribution. Above the connection points is the location for applying soluble fertilizers.

For each RF plot, a trench (8 m long × 30 cm wide × 30 cm deep) was excavated parallel to the tea row, centered within the inter-row space. Rockwool blocks (8 m long × 30 cm wide × 15 cm high) were fabricated with a central 4 cm diameter groove. A perforated PVC pipe (4 cm diameter, 8 m long, with 10 cm spacing between holes) was placed within the groove, and the rockwool block was closed around it to fully encapsulate the pipe. This assembly was then placed in the trench and leveled (Figure 7).

A gravity-fed system, consisting of an elevated PVC water storage tank (2 m diameter × 1.5 m height, 4.7 m^3^ capacity), supplied water to the plots. The main pipeline from the tank was connected to the perforated auxiliary pipe in each plot via a control valve and a fertilizer inlet. During fertilization, a pre-weighed, soluble fertilizer was added to the inlet. Opening the valve allowed water to dissolve and transport the fertilizer through the pipe, where it was absorbed by the surrounding rockwool and subsequently released into the adjacent 10–30 cm soil layer. The conventional surface fertilization treatment (CK) involved conventional surface broadcast fertilization. In each control plot, the same type and amount of fertilizer were uniformly applied to the soil surface, synchronized with the RF fertilization schedule.

### 4.3. Soil N_2_O Flux Measurement

Soil N_2_O fluxes were measured using the static chamber–gas chromatography technique [39]. After the installation of the RF system, chambers (50 cm × 50 cm × 50 cm) were buried to a depth of 10 cm in the soil. Three replicate chambers were installed in both the CK and RF zones within the same tea row. During the first week after fertilization, N_2_O fluxes were monitored daily until they declined to baseline levels, and subsequently, samples were taken once or twice a month. Gas samples were collected from the chamber headspace into vacuum-sealed gas sampling bags between 9:00 and 11:00 am to minimize diurnal variability. Samples were collected at 0, 10, 20, and 30 min after chamber closure. Detailed procedures for gas sampling are provided in a previous study [28]. All gas samples were analyzed within 12 h using gas chromatography (Agilent 7890A, Inc., Santa Clara, CA, USA) equipped with an electron capture detector (ECD). The N_2_O fluxes were determined from the rate of change in headspace N_2_O concentration using a nonlinear fitting approach, and sample sets with a coefficient of determination (r^2^) < 0.90 were rejected [39]. Mean fluxes and standard deviations were calculated for each treatment from six replicated plots using the following equation:

$$F=\rho \frac{V}{A}\frac{P}{P_{0}}\frac{T_{0}}{T}\frac{dC_{t}}{dt}$$

where F is the N_2_O flux (μg m^−2^ h^−1^); ρ denotes the N_2_O density under standard conditions (μg m^−3^); V is the chamber volume (m^3^); A denotes soil emission area (m^2^); P/P_0_ is the ratio of ambient pressure to standard pressure (MPa); T_0_/T denotes the ratio of standard temperature to sampling temperature (K); $dC_{t}/dt$ is the rate of N_2_O concentration change (μg m^−2^ h^−1^).

Cumulative N_2_O emissions were calculated by linearly interpolating fluxes between consecutive sampling dates:

$$M_{g}=\sum [\left(\frac{R_{i+1}+R_{i}}{2}\right)\times \left(t_{i+1}-t_{i}\right)]\times 24\times 10^{-5}$$

where M_g_ represents cumulative N_2_O emission (kg ha^−1^ yr^−1^); R_i_ (μg m^–2^ h^–1^) represents N_2_O flux at the ith sampling date; t_i_ refers to the day of the ith sampling (days).

Soil temperature and water content were measured at 10 cm depth near each chamber during gas sampling. The water-filled pore space (WFPS) was calculated based on the moisture content using the following formula:

$$WFPS=\frac{WC\times BD}{1-\frac{BD}{\rho }}\times 100\%$$

where WC represents mass water content (g g^−1^ dry soil); BD represents the soil bulk density (g cm^−3^); ρ represents particle density (2.37 g cm^−3^) [40].

### 4.4. Measurement of Tea Plant Biomass, Bud Yield and Bud Quality Indices

Tea yield was determined using the hundred-bud weight method. Three 50 cm × 50 cm quadrats were established per treatment. Within each quadrat, 100 buds meeting the plucking standard (one bud with one leaf or one bud with two leaves) were collected and weighed to determine the hundred-bud weight. The fresh leaves were enzyme-inactivated in an oven at 105 °C for 30 min, and then dried at 65 °C to constant weight. The dried tea leaves were weighed and pulverized using a ball mill, then stored for subsequent analysis.

Amino acid content in tea leaves was determined using the ninhydrin colorimetric method [41]. A 1.0 mL aliquot of the sample extract was mixed with 0.5 mL of phosphate buffer and 0.5 mL of 2% ninhydrin solution. The mixture was heated in a boiling water bath for 15 min, cooled to room temperature, diluted to 25.0 mL with distilled water, and the absorbance was measured at 570 nm to determine the amino acid concentration. Tea polyphenol content was determined using the ferric tartrate colorimetric method. A 1.0 mL aliquot of the sample extract was mixed with 4 mL of distilled water and 5 mL of ferric tartrate solution. After thorough mixing, the solution was diluted to 25.0 mL with phosphate buffer (pH 7.5), and the absorbance was measured at 540 nm to determine the tea polyphenol concentration. Total N, phosphorus (P), and potassium (K) contents in tea leaves were determined using the Kjeldahl method, the molybdenum blue colorimetric method, and flame photometry, respectively [41,42].

### 4.5. Soil Sampling and Analysis

Throughout the tea-growing season, soil samples were collected at three depths (0–10 cm, 10–20 cm, and 20–40 cm) on 27 July 2022, 31 October 2022 and 24 February 2023. At each sampling event, five soil cores were obtained from each depth near each static chamber and homogenized to form a single composite sample per layer. Composite samples were collected from six replicate plots per treatment. After removing plant residues and small gravel, the samples were sieved through a 2 mm mesh. The sieved soil was then divided into three subsamples: one was stored at 4 °C for microbial activity analysis; a second was snap-frozen in liquid N_2_ and stored at −80 °C for DNA extraction; and the third was air-dried in the shade for soil property analyses.

### 4.6. Soil Chemical and Microbial Analyses

Soil pH was measured in a 1:2.5 (*w*/*v*) soil-to-water suspension using a glass electrode pH meter (Five Easy Plus, Mettler Toledo, Nänikon, Switzerland). Soil organic carbon (SOC) and TN were determined by dry combustion using an elemental analyzer (Vario EL cube, Elementar, Hanau, Germany). Dissolved organic C (DOC) was determined by ultraviolet-enhanced persulfate digestion and infrared detection (Phoenix 8000, Teledyne Tekmar, Cincinnati, OH, USA) [43]. Microbial biomass C (MBC) and MBN were determined by the chloroform fumigation-extraction method, with extracts analyzed on a TOC analyzer (multi N/C 3100, Analytik, Jena, Germany). Soil inorganic N (NH_4_^+^-N and NO_3_^−^-N) was extracted with 2 M KCl; NH_4_^+^-N was measured by the indophenol blue method, and NO_3_^−^-N was measured by dual-wave length spectrophotometry at 220 and 275 nm (Spectrophotometer, Shimadzu UV-2600, Kyoto, Japan) [43,44].

### 4.7. DNA Extraction and Quantitative PCR

Soil DNA was extracted from 0.25 g of freeze-dried soil using the DNeasy^®^ PowerSoil^®^ Pro Kit (QIAGEN, Hilden, Germany) according to the manufacturer’s protocol. DNA concentration and purity were assessed using a Quick Drop spectrophotometer (Molecular Devices, San Jose, CA, USA), and high-quality extracts were stored at −40 °C. The abundances of N-cycling functional genes were quantified using a StepOnePlus Real-Time PCR System (Applied Biosystems, Carlsbad, CA, USA). Each 20 μL reaction contained 10 μL of SYBR^®^ Green Premix Ex Taq, 0.4 μL of ROX reference dye (50×), 0.4 μM each of forward and reverse primers, 2 μL of template DNA (10 ng μL^−1^), and 6.8 μL of nuclease-free water. The specificity of the amplification was confirmed by melting curve analysis. Primer sequences and thermal cycling conditions are listed in Table 4.

### 4.8. Statistical Analysis

Prior to statistical analysis, the Shapiro–Wilk and Levene’s test were used to evaluate the normality and homogeneity of variances. The data met the assumptions of normality and homogeneity of variances without transformation (Shapiro–Wilk test, *p* > 0.05; Levene’s test, *p* > 0.05). A one-way ANOVA was conducted to assess the impact of the RF treatment on soil properties, functional gene abundances, and N_2_O emissions. Means were compared using Fisher’s least significant difference (LSD) test when the ANOVA indicated significant differences. Pearson correlation analysis was carried out to investigate the relationships between N_2_O emissions, soil parameters, and gene abundances. Random forest analysis was performed using the ‘randomForest’ and ‘rfPermute’ packages in R (v 4.2.1). The importance of each factor was assessed using the percentage increase in mean squared error (MSE), with higher MSE values indicating greater significance. Results are presented as mean ± standard deviation (SD, *n* = 6). Statistical significance was determined at *p* < 0.05.

## 5. Conclusions

This study demonstrates that rockwool-based fertigation (RF) is an effective strategy for mitigating N_2_O emissions and promoting tea growth in plantations. And the RF system showed long-term sustainability in the sustained mitigation of N_2_O emissions. Thus, our results provide strong evidence that RF effectively achieves both nitrogen conservation and emission reduction in tea plantations, rendering it particularly well-suited for promotion in the southern hilly regions of China and other arid or semi-arid agricultural areas. This technology not only exhibits considerable application potential in tea plantations but also demonstrates comparable utility across the majority of orchards and economic forestlands. Future research should incorporate a gradient of fertilization rates to optimize the balance between agronomic performance and N_2_O mitigation, and long-term monitoring is warranted to fully capture the enduring effects of RF on soil biogeochemical processes.

## Figures and Tables

**Table 1:** The pruning, yield and quality of tea buds.

Fertilization Mode	Pruning (t ha^−1^)	Tea Yield (g)	Amino Acid (mg g^−1^)	Tea Polyphenol (mg g^−1^)	TN (mg g^−1^)	TP (mg g^−1^)	TK (mg g^−1^)
RF	10.59 ± 0.34 a	46 ± 0.8 a	51.31 ± 5.3 a	320.75 ± 8.7 a	38.62 ± 0.6 a	4.73 ± 0.2 a	18.73 ± 0.3 a
CK	9.87 ± 0.20 b	44 ± 1.5 a	45.53 ± 3.9 a	307.83 ± 10.6 a	37.97 ± 0.3 a	4.65 ± 0.1 a	18.41 ± 0.1 a

RF, rockwool-based fertigation; CK, conventional surface fertilization treatment; TN, total nitrogen; TP, total phosphorus; and TK, total potassium. Different lowercase letters indicate significant differences (*p* < 0.05).

**Table 2:** Effect of rockwool-based fertigation on the soil properties.

Sampling Time	Soil Properties	0–10 cm		10–20 cm		20–40 cm	
		RF	CK	RF	CK	RF	CK
27 July 2022	pH	4.32 ± 0.04	4.23 ± 0.03	4.30 ± 0.02	4.33 ± 0.02	4.32 ± 0.04	4.41 ± 0.02
	SOC (mg g^−1^)	20.47 ± 0.09	20.75 ± 0.31	11.77 ± 0.15	10.47 ± 0.15	9.16 ± 0.71	7.50 ± 0.08
	TN (mg g^−1^)	1.34 ± 0.03	1.60 ± 0.04	1.18 ± 0.03	1.04 ± 0.06	1.07 ± 0.07	0.81 ± 0.02
	C/N	15.30 ± 0.39	12.97 ± 0.38	9.95 ± 0.58	10.08 ± 0.28	8.55 ± 0.09	9.31 ± 0.28
	NH_4_^+^-N (mg kg^−1^)	20.49 ± 0.91	30.07 ± 1.83	14.36 ± 0.45	9.19 ± 0.22	10.91 ± 0.32	12.36 ± 0.45
	NO_3_^−^-N (mg kg^−1^)	58.86 ± 4.48	72.69 ± 2.32	26.73 ± 1.23	19.30 ± 1.46	14.50 ± 1.04	21.03 ± 1.07
	MBC (mg kg^−1^)	304.67 ± 4.80	391.69 ± 13.95	318.78 ± 3.52	301.89 ± 5.89	258.44 ± 5.63	203.10 ± 5.86
	MBN (mg kg^−1^)	25.24 ± 1.63	29.45 ± 2.20	20.97 ± 1.52	11.34 ± 1.49	17.15 ± 1.37	7.38 ± 1.06
	DOC (mg kg^−1^)	293.88 ± 6.78	314.10 ± 17.84	211.83 ± 20.37	244.17 ± 7.17	211.84 ± 8.25	165.24 ± 9.72
31 October 2022	pH	4.12 ± 0.03	3.83 ± 0.04	4.08 ± 0.02	4.10 ± 0.02	4.03 ± 0.02	4.09 ± 0.02
	SOC (mg g^−1^)	19.79 ± 0.23	20.54 ± 0.34	12.25 ± 0.14	9.93 ± 0.39	9.50 ± 0.07	8.05 ± 0.07
	TN (mg g^−1^)	1.40 ± 0.02	1.57 ± 0.02	1.21 ± 0.04	1.00 ± 0.02	1.04 ± 0.05	0.80 ± 0.01
	C/N	14.15 ± 0.30	13.11 ± 0.22	10.09 ± 0.32	9.95 ± 0.48	9.12 ± 0.41	10.12 ± 0.07
	NH_4_^+^-N (mg kg^−1^)	16.82 ± 1.38	34.04 ± 1.79	19.64 ± 0.81	10.61 ± 0.87	17.88 ± 2.14	9.28 ± 0.66
	NO_3_^−^-N (mg kg^−1^)	52.57 ± 6.03	79.92 ± 4.60	31.46 ± 2.64	21.30 ± 2.57	21.70 ± 1.81	20.00 ± 1.70
	MBC (mg kg^−1^)	296.17 ± 10.23	326.67 ± 9.91	328.50 ± 9.65	313.00 ± 8.97	275.67 ± 11.64	214.83 ± 13.08
	MBN (mg kg^−1^)	16.63 ± 1.40	26.83 ± 2.70	25.39 ± 2.46	15.49 ± 1.17	19.42 ± 1.80	8.31 ± 2.47
	DOC (mg kg^−1^)	306.4 ± 13.67	319.28 ± 12.32	257.29 ± 11.11	271.32 ± 8.49	209.90 ± 6.97	186.51 ± 12.14
24 February 2023	pH	4.15 ± 0.04	4.01 ± 0.05	4.19 ± 0.04	4.26 ± 0.03	4.30 ± 0.04	4.35 ± 0.05
	SOC (mg g^−1^)	19.74 ± 0.26	20.62 ± 0.14	12.56 ± 0.19	10.12 ± 0.11	9.10 ± 0.17	7.91 ± 0.17
	TN (mg g^−1^)	1.31 ± 0.04	1.60 ± 0.04	1.30 ± 0.03	1.00 ± 0.01	1.00 ± 0.02	0.77 ± 0.04
	C/N	15.10 ± 0.60	12.87 ± 0.41	9.70 ± 0.20	10.11 ± 0.14	9.08 ± 0.03	10.32 ± 0.56
	NH_4_^+^-N (mg kg^−1^)	9.45 ± 2.04	41.33 ± 9.45	27.79 ± 4.19	14.91 ± 1.40	94.43 ± 6.95	68.24 ± 7.68
	NO_3_^−^-N (mg kg^−1^)	45.48 ± 9.22	89.23 ± 11.29	40.41 ± 3.81	22.06 ± 2.83	34.17 ± 1.91	30.07 ± 2.57
	MBC (mg kg^−1^)	565.85 ± 17.70	667.69 ± 23.84	524.92 ± 18.37	438.74 ± 16.23	444.21 ± 10.77	412.46 ± 10.44
	MBN (mg kg^−1^)	38.69 ± 3.06	49.51 ± 4.31	40.77 ± 2.74	16.09 ± 1.74	33.71 ± 3.28	7.71 ± 1.36
	DOC (mg kg^−1^)	298.08 ± 12.75	355.12 ± 12.90	303.45 ± 8.70	332.87 ± 13.20	257.03 ± 12.02	227.53 ± 13.60

SOC, soil organic carbon; TN, total nitrogen; C/N, ratio of soil organic carbon to total nitrogen; MBC, microbial biomass carbon; MBN, microbial biomass nitrogen; and DOC, dissolved organic carbon.

**Table 3:** Basic values of soil physicochemical properties in experimental plots (mean ± SD, *n* = 12).

Soil Depth	pH	SOC (g/kg)	TN (g/kg)	C/N	NH_4_^+^-N (mg/kg)	NO_3_^−^-N (mg/kg)
0–10 cm	4.09 ± 0.03	20.07 ± 0.16	1.34 ± 0.02	14.93 ± 0.21	32.04 ± 1.33	80.14 ± 2.77
10–20 cm	4.23 ± 0.05	10.49 ± 0.62	0.98 ± 0.03	10.68 ± 0.78	16.43 ± 0.57	63.67 ± 4.60
20–40 cm	4.39 ± 0.05	8.10 ± 0.29	0.86 ± 0.02	9.46 ± 0.50	20.34 ± 3.05	50.89 ± 2.38

**Table 4:** Primers for amplification of functional genes in this study.

Genes	Primer Set	Sequence (5′-3′)	Thermal Profile	Reference
AOA	CrenamoA23f	ATGGTCTGGCTWAGACG	30 s-95 °C, 95 °C-15 s, 55 °C-30 s, 72 °C-30 s, 80 °C-30 s	[45]
	CrenamoA616r	GCCATCCATCTGTATGTCCA	95 °C-5 s, 57 °C-34 s, 72 °C-15 s, 95 °C-15 s, 55 °C-30 s, 72 °C-30 s, 80 °C-30 s	
AOB	*amoA*-1F	GGGGTTTCTACTGGTGGT	30 s-95 °C, 95 °C-15 s, 55 °C-30 s, 72 °C-30 s, 80 °C-30 s	[46]
	*amoA*-2R	CCCCTCKGSAAAGCCTTCTTC	95 °C-5 s, 55 °C-34 s, 72 °C-15 s, 95 °C-15 s, 55 °C-30 s, 72 °C-30 s, 80 °C-30 s	
*nirK*	*nirK*-F1aCu	ATCATGGTSCTGCCGCG	30 s-95 °C, 95 °C-15 s, 55 °C-30 s, 72 °C-30 s, 80 °C-30 s	[47]
	*nirK*-R3Cu	GCCTCGATCAGRTTGTGGTT	95 °C-5 s, 58 °C-34 s, 72 °C-15 s, 95 °C-15 s, 55 °C-30 s, 72 °C-30 s, 80 °C-30 s	
*nirS*	*nirS*-Cd3aF	TACCACCCSGARCCGCGCGT	30 s-95 °C, 95 °C-15 s, 55 °C-30 s, 72 °C-30 s, 80 °C-30 s	[48]
	*nirS*-R3cd	GCCGCCGTCRTGVAGGAA	95 °C-5 s, 58 °C-34 s, 72 °C-15 s, 95 °C-15 s, 55 °C-30 s, 72 °C-30 s, 80 °C-30 s	
*nosZ*	*nosZ*-F	AGAACGACCAGCTGATCGACA	30 s-95 °C, 95 °C-15 s, 55 °C-30 s, 72 °C-30 s, 80 °C-30 s	[49]
	*nosZ*-R	TCCATGGTGACGCCGTGGTTG	95 °C-5 s, 60 °C-34 s, 72 °C-15 s, 95 °C-15 s, 55 °C-30 s, 72 °C-30 s, 80 °C-30 s	

## Data Availability

The data were shared by a dataset.

## Supplementary Materials

The following supporting information can be downloaded at: https://www.mdpi.com/article/10.3390/plants15121862/s1, Figure S1: Dependence of N_2_O fluxes on soil temperature; Figure S2: Dependence of N_2_O fluxes on WFPS; Figure S3 Effect of Rockwool-based fertigation on proportion of reduction in cumulative N_2_O emissions; Figure S4 Effect of Rockwool-based fertigation on the abundance of nitrification genes; Figure S5 Effect of Rockwool-based fertigation on the abundance of denitrification genes.
