# Supplementary material for: Rockwool-Based Fertigation Enhances Tea Plant Growth While Mitigating Soil N2O Emissions
Source: Plants (Basel). 2026 Jun 16;15(12):1862. doi: 10.3390/plants15121862 (PMC13306667; doi:10.3390/plants15121862)
Supplement: Supplementary file 1 [file plants-15-01862-s001.zip › plants-4324336-supplementary.pdf]

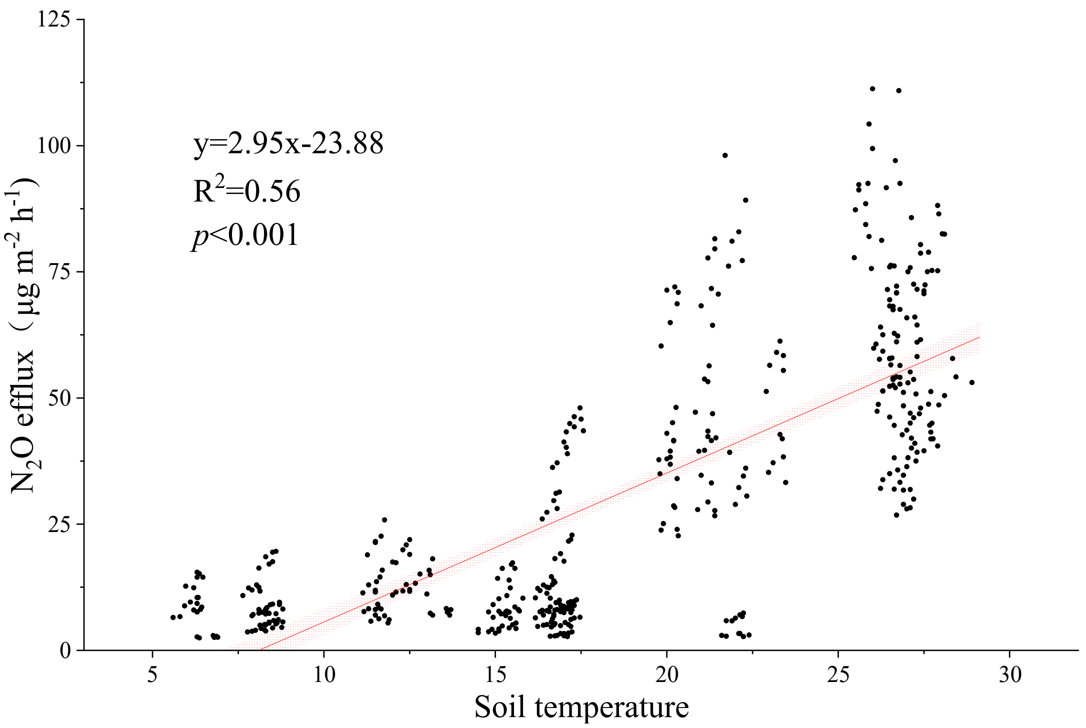

Supplemental Figure S1 Dependence of N_2O fluxes on soil temperature

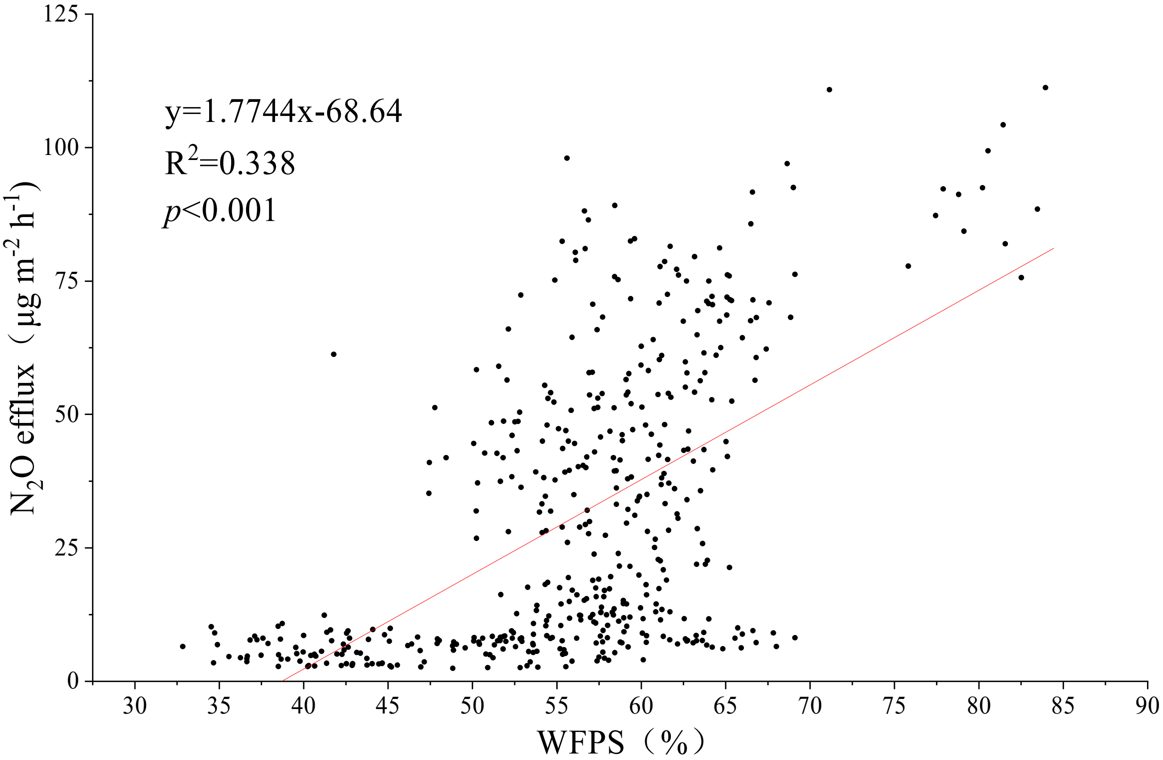

Supplemental Figure S2 Dependence of N_2O fluxes on WFPS

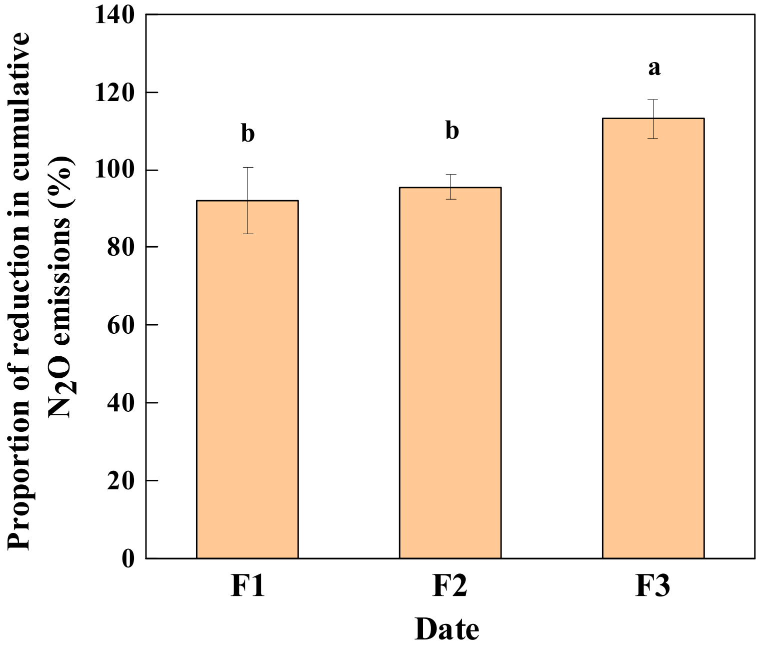

Supplemental Figure S3 Effect of Rockwool-based fertigation on proportion of reduction in cumulative N_2O emissions (%).

F1: The first 90 days after fertilization from October 31, 2021 to January 26, 2022. F2: The second 90 days after fertilization from July 27, 2022 to October 24, 2022. F3: The second 90 days after fertilization from October 31, 2022 to January 28, 2023. Different capital letters indicate significant differences among different 90 days after fertilization. Error bars represent standard errors of the mean (SE, $n = 6$).

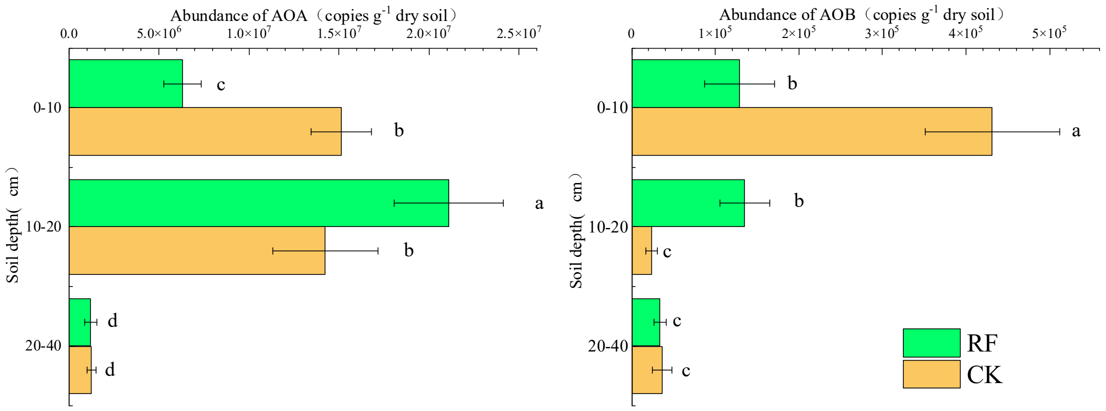

Supplemental Figure S4 Effect of Rockwool-based fertigation on the abundance of nitrification genes

CK: conventional surface fertilization; RF: Rockwool-based fertigation.

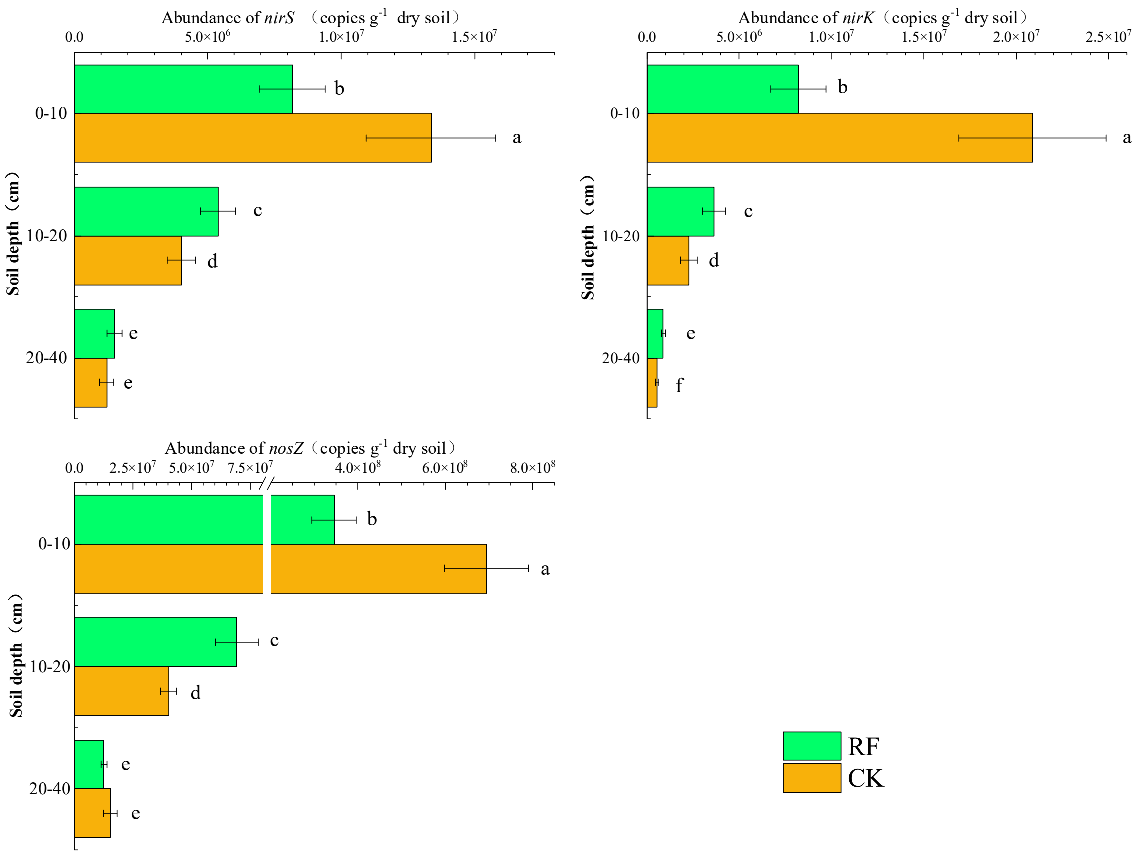

Supplemental Figure S5 Effect of Rockwool-based fertigation on the abundance of denitrification genes

CK: conventional surface fertilization; RF: Rockwool-based fertigation.
